# Factors associated with initial or subsequent choice of biologic disease-modifying antirheumatic drugs for treatment of rheumatoid arthritis

**DOI:** 10.1186/s13075-017-1366-1

**Published:** 2017-07-05

**Authors:** Yinzhu Jin, Rishi J. Desai, Jun Liu, Nam-Kyong Choi, Seoyoung C. Kim

**Affiliations:** 1 Division of Pharmacoepidemiology and Pharmacoeconomics, Brigham and Women’s Hospital, 1620 Tremont Street, Suite 3030, Boston, MA 02120 USA; 2 Institute of Environmental Medicine, Medical Research Center, Seoul National University, Seoul, Republic of Korea; 3 Department of Health Convergence, Ewha Womans University, Seoul, Republic of Korea; 4 Division of Rheumatology, Immunology and Allergy, Brigham and Women’s Hospital, Boston, MA USA

**Keywords:** Rheumatoid arthritis, Antirheumatic agents, Biologic therapy

## Abstract

**Background:**

Biologic disease-modifying antirheumatic drugs (DMARDs) are increasingly used for rheumatoid arthritis (RA) treatment. However, little is known based on contemporary data about the factors associated with DMARDs and patterns of use of biologic DMARDs for initial and subsequent RA treatment.

**Methods:**

We conducted an observational cohort study using claims data from a commercial health plan (2004–2013) and Medicaid (2000–2010) in three study groups: patients with early untreated RA who were naïve to any type of DMARD and patients with prevalent RA with or without prior exposure to one biologic DMARD. Multivariable logistic regression models were used to examine the effect of patient demographics, clinical characteristics and healthcare utilization factors on the initial and subsequent choice of biologic DMARDs for RA.

**Results:**

We identified a total of 195,433 RA patients including 78,667 (40%) with early untreated RA and 93,534 (48%) and 23,232 (12%) with prevalent RA, without or with prior biologic DMARD treatment, respectively. Patients in the commercial insurance were 87% more likely to initiate a biologic DMARD versus patients in Medicaid (OR = 1.87, 95% CI = 1.70–2.05). In Medicaid, African-Americans had lower odds of initiating (OR = 0.59, 95% CI = 0.51–0.68 in early untreated RA; OR = 0.71, 95% CI = 0.61–0.74 in prevalent RA) and switching (OR = 0.71, 95% CI = 0.55–0.90) biologic DMARDs than non-Hispanic whites. Prior use of steroid and non-biologic DMARDs predicted both biologic DMARD initiation and subsequent switching. Etanercept, adalimumab, and infliximab were the most commonly used first-line and second-line biologic DMARDS; patients on anakinra and golimumab were most likely to be switched to other biologic DMARDS.

**Conclusions:**

Insurance type, race, and previous use of steroids and non-biologic DMARDs were strongly associated with initial or subsequent treatment with biologic DMARDs.

## Background

Rheumatoid arthritis (RA) is a common autoimmune inflammatory arthritis affecting over 1.3 million people in the USA [1]. Treatment with disease-modifying antirheumatic drugs (DMARDs) is considered the standard of care for RA [2, 3]. The 2012 American College of Rheumatology (ACR) guidelines recommend monotherapy with a non-biologic DMARD or double and triple therapy for early-stage RA [2]. Adding or switching to a tumor necrosis factor (TNF) inhibitor, abatacept, or rituximumab is recommended for patients who still experience moderate or high disease activity after 3 months of non-biologic DMARD treatment. If the initial biologic DMARD does not result in adequate response or causes adverse events, switching to another TNF inhibitor or non-TNF biologic drug is recommended [2].

Over the past two decades, major advances have occurred in the treatment of RA with development of novel biologic drugs targeting specific components of the immune system. A prior study based on a prospective RA cohort in California showed a remarkable change over the past two decades in the use of DMARDs in patients with longstanding RA. The proportion of patients receiving any biologic DMARD increased from 10 to 48% between 1999 and 2009 [4]. The rising use of biologic drugs in RA in clinical practice as more drugs have become available supports a need for examining the characteristics of patients who receive biologic treatment and the relationship between the patients’ characteristics and the biologic treatment. A number of biologic DMARDs are currently available for treatment of RA including five TNF inhibitors (adalimumab, certolizumab, etanercept, golimumab, and infliximab) and four non-TNF inhibitors (abatacept, anakinra, rituximab, and tocilizumab). Tofacitinib is an oral Janus kinase inhibitor for the treatment of RA, recently approved by the US Food and Drug Administration (FDA) in 2012 for treatment of RA with moderate to severe disease activity.

As recommended by ACR guidelines, the decision to start or switch a biologic DMARD should be made based on a patient’s disease activity, prior DMARD treatment, and comorbid conditions [2]. Current evidence on predictors of biologic treatment in RA patients varies across studies. Some studies have demonstrated that initiation of biologic treatment is mainly associated with disease-related and treatment-related factors such as prior use of glucocorticoids, non-biologic DMARDs, or comorbidities [5, 6]. Several studies have highlighted sociodemographic disparities in access to biologic DMARDs [5, 7, 8]. Older age was considered to be negatively associated with biologic treatment in all of these studies. These previous studies vary in terms of study period, RA disease stage, and population, leading to discrepancies in the conclusions. Furthermore, previous studies have only focused on initiation of biologic DMARDs and there is no evidence on switching biologic DMARDs as an outcome. In addition, little work has been done based on contemporary population-based data to describe the patterns of use of different biologic DMARDs for initial and subsequent RA treatment. Therefore, we conducted a cohort study using claims data from two large insurance programs from the USA to describe factors associated with initial and subsequent choice of biologic DMARD and the patterns of treatment sequence among various biologic DMARDs for the treatment of RA over 12 years.

## Methods

### Data source

We used the claims data from the US commercial health plan, United Healthcare (2004–2013), and a public health plan, Medicaid Analytic eXtract (MAX, 2000–2010). The United Healthcare plan insures primarily working adults and their family members across the USA, and Medicaid is a joint federal and state program that helps low-income individuals and families with the costs associated with medical and long-term custodial care. Both databases contain longitudinal information on pharmacy dispensing, medical diagnoses, procedures, hospitalizations, and physician visits across the USA. The study protocol was approved by the Institutional Review Board of the Brigham and Women’s Hospital. Personal identifiers were removed from the dataset before the analysis to protect subject confidentiality. Patient informed consent was therefore not required.

### Study cohort

**Fig. 1:**
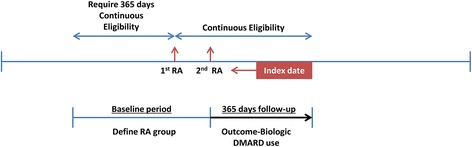
Definition of the cohort. Eligible patients were required to have two diagnoses of rheumatoid arthritis (*RA*) that were ≥7 days but <365 days apart, with continuous insurance coverage between one year prior to the first RA diagnosis (*1* ^*st*^ *RA*) date and one year after the second RA diagnosis (*2* ^*nd*^ *RA*) date. The second RA diagnosis date was defined as the index date, and the baseline period was defined as time between the second RA diagnosis and one year prior to the first RA diagnosis

We identified patients aged ≥18 years with two diagnoses of RA (International Classification of Disease, Ninth Revision, ICD-9 diagnosis code 714.xx) that were ≥7 days but <365 days apart. Eligible patients were required to have continuous insurance coverage between one year prior to the first RA diagnosis date and one year after the second RA diagnosis date. The second RA diagnosis date was defined as the index date, and the baseline period was defined as the time between the second RA diagnosis and one year prior to the first RA diagnosis (see Fig. 1). We measured baseline DMARD use by identifying prescription fills of 21 individual non-biologic and biologic DMARDs. Non-biologic DMARDs were methotrexate (MTX), hydroxychloroquine (HCQ), sulfasalazine, leflunomide, gold compounds, mycophenolate mofetil, penicillamine, minocycline, azathioprine, cyclophosphamide, and cyclosporine. Biologic DMARDs included five TNF inhibitors (etanercept, adalimumab, certolizumab, golimumab, and infliximab), and four non-TNF biologic drugs (abatacept, anakinra, rituximab, and tocilizumab). We also included tofacitinib in the biologic group although this is a unique class of DMARD.

We separated the identified cohort into three mutually exclusive groups based on the stage of RA and use of biologic DMARDs during the baseline period. Group 1 was defined as patients with early untreated RA who had only one RA diagnosis in the baseline period and did not use any DMARDs during this time; group 2 was defined as patients with prevalent RA who were naïve to biologic DMARDs but had used at least one non-biologic DMARD or had received more than one RA diagnosis in the baseline period; and group 3 was defined as patients with prevalent RA who had already used a single biologic DMARD during the baseline period.

### Study outcomes

During the 12-month follow-up period, we identified initiation of the first-ever biologic DMARD among patients with early untreated RA, initiation of the first-ever biologic agent among biologic-DMARD-naïve patients with prevalent RA, and initiation of a second biologic agent among biologic-exposed patients with prevalent RA. The outcomes of the study were defined as binary variables (yes or no) indicating whether initiation or switching (initiation of a second biologic) of a biologic DMARD occurred.

### Predictors of interest

Variables potentially related to biologic treatment decisions were assessed in the baseline period. First, we selected variables that indicate RA disease activity. These variables included prior use of steroids (groups 1–3), non-biologic DMARDs (groups 2–3), and biologic DMARDs for those already on a biologic agent (group 3). Underlying comorbid conditions and medication history that may affect choice of biologic treatment were chosen as potential predictors, which included hyperlipidemia, heart disease, hypertension, cerebrovascular accident, diabetes mellitus, obesity, psoriatic arthritis, ankylosing spondylitis, inflammatory bowel disease, history of hospitalization with severe infection, chronic obstructive pulmonary disease (COPD), liver disease, metastatic cancer, tumor, alcohol abuse, tobacco use, a combined comorbidity score [9], use of angiotensin-converting enzyme (ACE) inhibitors, angiotensin II receptor blockers (ARBs), beta blockers, calcium channel blockers, diuretics, insulin, oral hypoglycemic drugs, statins, non-statin lipid lowering drugs, aspirin, opioids, cyclooxygenase inhibitors (coxibs), and non-selective non-steroidal anti-inflammatory drugs (NSAIDs). Heart disease was defined as acute myocardial infarction, angina, chronic heart failure, or other forms of chronic ischemic heart disease. Liver disease was defined as chronic hepatitis, chronic liver disease and cirrhosis, other sequelae of chronic liver disease, or liver transplant. The combined comorbidity score is a single numerical score that combines 20 elements from the Charlson index and the Elixhauser system to summarize patients’ co-morbid conditions [9]. We also included healthcare utilization factors to account for general patient health and contact with the healthcare system. These factors included the number of total distinct medications dispensed, number of physician visits, number of hospitalizations, and number of emergency room visits in the 1-year period prior to the index date. Finally, to identify predictors reflecting potential disparities in receipt of biologic DMARD treatment, we selected the insurance program (United Healthcare or Medicaid), age, gender, calendar year, region, and race/ethnicity (only available in Medicaid).

### Statistical analysis

To estimate association between potential predictors and biologic initiation/switch, we applied multivariable logistic regression in each of the three cohort groups. Baseline covariates that we identified were all included in each model. In addition, in the models in the prevalent RA groups (groups 2 and 3) we also included the use of MTX, HCQ, and total number of different non-biologic DMARD prescriptions during the prior year. For group 3 we additionally included prior use of biologic DMARDs categorized by their generic names.

As we had information on patients’ race and ethnicity only in the Medicaid database, we conducted an additional analysis to examine the racial/ethnicity disparities in biologic DMARD use. Some Medicaid patients also would be eligible for the Medicare plan (i.e., dual-eligible patients). Since these patients may have different coverage for biologic agents than those with only Medicaid, we conducted sensitivity analysis by excluding Medicaid patients who also received Medicare coverage at any time during the entire study period. We applied the same aforementioned multivariable logistic model for this sensitivity analysis in each of the three different RA groups. All analyses were performed using SAS version 9.4.

## Results

### Baseline characteristics

A total of 195,433 patients were identified: 82,402 (42.2%) from the United Healthcare and 113,031(57.8%) from the Medicaid database. The mean age was 49 (±12) years and 79% were female. There were 78,667 patients with early untreated RA who did not have DMARD treatment before the index date. Among patients with prevalent RA there were 93,534 and 23,232 without or with prior biologic DMARD treatment, respectively (Table 1). Of the patients with early untreated RA, 66% were enrolled in Medicaid, whereas 67% of patients with prevalent RA who already initiated biologic DMARDs were enrolled in the commercial insurance program.

**Table 1:** Baseline characteristics of the three cohorts

	All	Early untreated RA	Prevalent RA, naïve to biologic DMARDs	Prevalent RA, exposed to biologic DMARDs
Total number	195,433 (100)	78,667 (100)	93,534 (100)	23,232 (100)
Insurance type				
Commercial (United)	82,402 (42.2)	26,810 (34.1)	40,023 (42.8)	15,569 (67.0)
Public (Medicaid)	113,031 (57.8)	51,857 (65.9)	53,511 (57.2)	7663 (33.0)
Age	49 (±12.1)	49 (±12.2)	50 (±12.1)	49 (±11.9)
Gender (female)	153,933 (78.8)	60,487 (76.9)	75,456 (80.7)	17,990 (77.4)
Comorbid conditions				
Hyperlipidemia	76,793 (39.3)	36,538 (46.4)	33,005 (35.3)	7250 (31.2)
Heart disease^a^	34,869 (17.8)	17,501 (22.2)	14,896 (15.9)	2472 (10.6)
Hypertension	92,080 (47.1)	41,338 (52.5)	42,244 (45.2)	8498 (36.6)
Cerebrovascular accident	10,118 (5.2)	5432 (6.9)	4175 (4.5)	511 (2.2)
Diabetes	41,704 (21.3)	20,195 (25.7)	18,143 (19.4)	3366 (14.5)
Obesity	22,828 (11.7)	12,317 (15.7)	8829 (9.4)	1682 (7.2)
Inflammatory bowel disease	4037 (2.1)	1475 (1.9)	1661 (1.8)	901 (3.9)
History of hospitalization with severe infections^b^	8876 (4.6)	4539 (5.8)	3689 (3.9)	648 (2.8)
COPD	62,388 (31.9)	30,789 (39.1)	26,974 (28.8)	4625 (19.9)
Liver disease	13,030 (6.7)	7035 (8.9)	5057 (5.4)	938 (4.0)
Metastatic cancer	1849 (1.0)	1028 (1.3)	719 (0.8)	102 (0.4)
Any tumor	12,976 (6.6)	6301 (8.0)	5683 (6.1)	992 (4.3)
Alcohol abuse	8699 (4.5)	5385 (6.8)	2928 (3.1)	386 (1.7)
Tobacco use	35,992 (18.4)	18,567 (23.6)	14,562 (15.6)	2863 (12.3)
Combined comorbidity score	1 (±2.0)	2 (±2.0)	1 (±1.9)	1 (±1.5)
History of medication use				
ACE inhibitors	35,742 (18.3)	15,088 (19.2)	17,107 (18.3)	3547 (15.3)
ARBs	16,311 (8.4)	7159 (9.1)	7310 (7.8)	1842 (7.9)
Beta blockers	30,934 (15.8)	12,955 (16.5)	14,679 (15.7)	3300 (14.2)
Calcium channel blockers	28,302 (14.5)	11,936 (15.2)	13,820 (14.8)	2546 (11.0)
Diuretics	52,688 (27.0)	22,093 (28.1)	25,279 (27.0)	5316 (22.9)
Insulin	8851 (4.5)	4100 (5.2)	3920 (4.2)	831 (3.6)
Oral hypoglycemic	21,004 (10.8)	95,28 (12.1)	9620 (10.3)	1856 (8.0)
Statins	38,246 (19.6)	173,91 (22.1)	17,079 (18.3)	3776 (16.3)
Non-statin lipid-lowering drugs	11,324 (5.8)	5213 (6.6)	4845 (5.2)	1266 (5.4)
Aspirins	10,876 (5.6)	58,42 (7.4)	4517 (4.8)	517 (2.2)
COX-2 inhibitors	44,555 (22.8)	150,28 (19.1)	24,130 (25.8)	5397 (23.2)
Non-selective NSAIDs	108,196 (55.4)	468,58 (59.6)	51,080 (54.6)	10,258 (44.2)
Opioids	118,542 (60.7)	485,45 (61.7)	56,545 (60.5)	13,452 (57.9)
Steroids	106,676 (54.6)	33,474 (42.6)	57,604 (61.6)	15,598 (67.1)
Cumulative steroid dose (mg/day in prior year)	3 (±32.8)	2 (±3.2)	4 (±43.3)	4 (±20.3)
Average daily steroid dose				
None	109, 365 (56.0)	53,489 (68.0)	45,373 (48.5)	10,503 (45.2)
Low (<5 mg/day)	66,779 (34.2)	22,933 (29.2)	35,157 (37.6)	8689 (37.4)
Medium (5–10 mg/day)	12,610 (6.5)	1473 (1.9)	8408 (9.0)	2729 (11.7)
High (≥10 mg/day)	6679 (3.4)	772 (1.0)	4596 (4.9)	1311 (5.6)
Non-biologic drugs	−	−	66,961 (71.6)	16,550 (71.2)
Number of non-biologic drugs	−			
None	−	−	26,573 (28.4)	6682 (28.8)
One	−	−	48,124 (51.5)	11,822 (50.9)
More than one	−	−	18,837 (20.1)	4728 (20.4)
Prior MTX	−	−	38,172 (40.8)	12,381 (53.3)
Prior HCQ	−	−	28,901 (30.9)	3735 (16.1)
Health care utilization				
Number of prescriptions	14 (±9.6)	14 (±10.2)	14 (±9.3)	13 (±8.2)
Number of physician visits	11 (±9.4)	11 (±9.5)	11 (±9.4)	12 (±8.4)
Number of hospitalizations	0 (±1.0)	0 (±1.2)	0 (±1.0)	0 (±0.7)
Number of emergency room visits	1 (±3.0)	1 (±3.6)	1 (±2.7)	1 (±1.7)

Values are the number (%) or mean (±SD). *RA* rheumatoid arthritis, *DMARD* disease-modifying antirheumatic drug, *COPD* chronic obstructive pulmonary disease, *ACE* angiotensin-converting enzyme, *ARB* angiotensin II receptor blocker, *COX* cyclooxygenase, *NSAID* non-selective non-steroidal anti-inflammatory drug, *MTX* methotrexate, *HCQ* hydroxychloroquine. ^a^Acute myocardial infarction, angina, chronic heart failure, and other forms of chronic heart disease. ^b^History of hospitalization with serious bacterial infections or opportunistic infections

The prevalence of comorbidities such as hyperlipidemia, hypertension, diabetes, and COPD was high (21–39%) across all cohort groups: 43% of the overall cohort had used steroids prior to their initiation of DMARD treatment, and more than 60% of patients with prevalent RA had used steroids before initiating a biologic DMARD. The average daily steroid dose was <5 mg/day in most patients who had steroids during the baseline period. The proportion of medium (5–10 mg/day) and high (≥10 mg/day) intake of steroids was higher in patients with prevalent RA than in patients with early untreated RA.

More than 70% of the patients with prevalent RA (both biologic-DMARD-naïve and prevalent users) had one non-biologic agent during their baseline period. One fifth of each of these two cohorts used more than one type of non-biologic DMARDs. Use of MTX, the most commonly used non-biologic DMARD, was common among both biologic-DMARD-naive patients with prevalent RA and those who had used a biologic DMARD before (40.8% and 53.3%, respectively, Table 1).

### Factors associated with biologic initiation and subsequent use

The multivariable logistic regression model (Table 2) showed strong association between the insurance type and the initiation of biologic DMARDs. The adjusted odds ratio (OR) of biologic DMARD initiation was 1.87 (95% CI = 1.70–2.05) in patients who were enrolled in the United Healthcare program compared to patients under Medicaid coverage. The sensitivity analysis after excluding patients with dual eligibility yielded very similar results to those from the main analysis (results not shown). Older age was associated with decreased odds of biologic DMARD prescription in all three cohorts. With a 10-year increase in age, the likelihood of initiating a biologic DMARD reduced by 13% in patients with early untreated RA and by 29% in those with prevalent RA. The odds of switching to a different biologic DMARD were also decreased by 13% per 10-year increase in age among patients with prevalent RA. Female patients were more likely to initiate or switch to a biologic DMARD than male patients.

**Table 2:** Adjusted odds ratio (95% CI) of initiation or switching biologic DMARDs

	Biologic DMARD initiation among patients with early untreated RA	Biologic DMARD initiation among patients with prevalent RA	Switching of biologic DMARDs among biologic DMARD users
Total number	78,667 (100%)	93,534 (100%)	23,232 (100%)
Biologic DMARD initiation/switch, *n*	3873 (4.9%)	10,361 (11.1%)	2761 (11.9%)
Data source			
United vs. Medicaid	1.87 (1.70, 2.05)	1.13 (1.06, 1.20)	0.92 (0.81, 1.05)
Age (by 10-year increase)	0.87 (0.84, 0.89)	0.81 (0.79, 0.83)	0.87 (0.84, 0.91)
Gender			
Male vs. female	0.90 (0.83, 0.98)	0.95 (0.89, 1.00)	0.87 (0.79, 0.97)
Comorbid conditions in prior year			
Hyperlipidemia	0.90 (0.83, 0.97)	0.97 (0.92, 1.03)	1.02 (0.91, 1.13)
Heart disease	0.92 (0.82, 1.03)	1.02 (0.94, 1.10)	1.12 (0.96, 1.31)
Hypertension	0.85 (0.78, 0.94)	0.91 (0.86, 0.97)	0.94 (0.83, 1.06)
Cerebrovascular accident	0.91 (0.76, 1.08)	0.94 (0.83, 1.06)	1.21 (0.92, 1.57)
Diabetes mellitus	0.93 (0.83, 1.04)	1.01 (0.93, 1.09)	0.95 (0.80, 1.12)
Obesity	1.02 (0.92, 1.12)	0.98 (0.91, 1.06)	0.99 (0.85, 1.16)
Inflammatory bowel disease	1.89 (1.56, 2.30)	1.22 (1.06, 1.42)	0.71 (0.56, 0.89)
Hospitalization with severe infection	0.82 (0.67, 1.00)	1.03 (0.91, 1.16)	0.86 (0.66, 1.11)
COPD	0.80 (0.74, 0.87)	0.97 (0.92, 1.02)	1.06 (0.95, 1.18)
Liver disease	1.16 (1.01, 1.33)	1.16 (1.05, 1.29)	1.03 (0.84, 1.27)
Metastatic cancer	0.92 (0.61, 1.39)	0.94 (0.68, 1.29)	0.29 (0.10, 0.81)
Any tumor	1.01 (0.87, 1.17)	0.93 (0.84, 1.03)	0.98 (0.79, 1.22)
Alcohol abuse	0.77 (0.64, 0.93)	0.94 (0.82, 1.09)	0.75 (0.53, 1.06)
Tobacco use	1.01 (0.92, 1.10)	1.13 (1.06, 1.20)	1.03 (0.92, 1.17)
Combined comorbidity score	0.96 (0.93, 0.98)	0.95 (0.94, 0.97)	1.01 (0.97, 1.05)
History of medication use in prior year			
Non-statin lipid-lowering drugs	1.10 (0.94, 1.27)	1.13 (1.02, 1.25)	1.04 (0.86, 1.25)
Statins	1.03 (0.93, 1.15)	1.02 (0.96, 1.10)	0.89 (0.77, 1.02)
ACE inhibitors	1.08 (0.97, 1.20)	1.00 (0.94, 1.07)	0.96 (0.84, 1.09)
ARBs	0.98 (0.85, 1.13)	1.10 (1.01, 1.20)	1.06 (0.90, 1.25)
Beta blockers	0.91 (0.81, 1.02)	0.91 (0.85, 0.97)	0.94 (0.83, 1.07)
Calcium channel blockers	0.88 (0.78, 0.99)	0.92 (0.86, 0.99)	0.90 (0.78, 1.04)
Diuretics	1.16 (1.06, 1.28)	1.01 (0.96, 1.08)	1.09 (0.97, 1.22)
Insulin	1.55 (1.28, 1.87)	1.36 (1.20, 1.53)	1.04 (0.81, 1.34)
Oral hypoglycemic drugs	1.23 (1.06, 1.43)	1.05 (0.95, 1.15)	0.98 (0.80, 1.20)
Aspirins	0.99 (0.82, 1.19)	0.83 (0.73, 0.94)	0.75 (0.55, 1.01)
Coxibs	1.46 (1.32, 1.61)	1.30 (1.23, 1.38)	1.11 (1.00, 1.23)
Non-selective NSAIDs	1.30 (1.20, 1.39)	1.07 (1.02, 1.12)	1.12 (1.03, 1.22)
Opioids	1.18 (1.09, 1.27)	1.19 (1.13, 1.25)	1.21 (1.10, 1.34)
Steroid daily dosage			
None	Reference	Reference	Reference
Low (<5 mg/day)	2.42 (2.25, 2.60)	1.65 (1.57, 1.73)	1.52 (1.38, 1.68)
Medium (5–10 mg/day)	3.12 (2.56, 3.80)	1.89 (1.76, 2.04)	1.53 (1.34, 1.75)
High (≥10 mg/day)	2.61 (1.91, 3.57)	1.72 (1.55, 1.89)	1.81 (1.51, 2.15)
Number of non-biologic DMARDs			
None	−	Reference	Reference
One	−	1.54 (1.42, 1.66)	1.44 (1.27, 1.64)
More than one	−	2.40 (2.17, 2.67)	1.98 (1.65, 2.36)
Prior MTX	−	1.79 (1.69, 1.90)	0.78 (0.70, 0.87)
Prior HCQ	−	0.57 (0.54, 0.61)	0.75 (0.65, 0.86)
Healthcare utilization in prior year			
Number of prescriptions	0.97 (0.97, 0.98)	0.99 (0.98, 0.99)	1.01 (1.00, 1.02)
Number of physician visits	1.00 (1.00, 1.01)	1.00 (1.00, 1.01)	1.01 (1.00, 1.01)
Number of hospitalizations	1.02 (0.97, 1.06)	1.05 (1.02, 1.08)	0.98 (0.92, 1.05)
Number of emergency room visits	0.98 (0.96, 1.00)	0.97 (0.95, 0.98)	1.00 (0.97, 1.02)

Odds ratios were adjusted for demographics, data source, calendar year, comorbidities, history of medication use, and healthcare utilization in the baseline period. *RA* rheumatoid arthritis, *DMARD* disease-modifying antirheumatic drug, *COPD* chronic obstructive pulmonary disease, *ACE* angiotensin-converting enzyme, *ARB* angiotensin II receptor blocker, *Coxib* Cyclooxygenase inhibitor, *NSAID* non-selective non-steroidal anti-inflammatory drug, *MTX* methotrexate, *HCQ* hydroxychloroquine

In the Medicaid cohort, we observed strong race disparities in biologic DMARD utilization. After adjusting for demographics, comorbidities, medication history, and health utilization characteristics, African-Americans were 30–40% less likely to initiate biologic DMARDs (OR = 0.59, 95% CI = 0.51–0.68 in the early untreated RA group; OR = 0.71, 95% CI = 0.61–0.74 in the prevalent RA group) or to switch biologic DMARDs in the prevalent RA group (OR = 0.71, 95% CI = 0.55–0.90) compared to the white, non-Hispanic population (Table 3).

**Table 3:** Race/ethnicity disparities in the use of biologic DMARDs among RA patients in Medicaid

Cohort group	Race/ethnicity	Total, *n*	Biologic DMARD initiation/switch, *n*	No biologic DMARD initiation/switch, *n*	Adjusted OR (95% CI)
Odds of biologic DMARD initiation^a^					
Early untreated RA, biologic-DMARD-naive group	White, non-Hispanic	23,627	794	22,833	Ref
	Black, non-Hispanic	13,531	275	13,256	0.59 (0.51, 0.68)
	Other non-Hispanic	4824	133	4691	0.88 (0.72, 1.06)
	Hispanic	9875	403	9472	1.24 (1.08, 1.42)
	Total	51,857	1605	50,252	−
Prevalent RA, biologic-DMARD-naive group	White, non-Hispanic	25,537	2570	22,967	Ref
	Black, non-Hispanic	11,171	759	10,412	0.71 (0.61, 0.74)
	Other non-Hispanic	5950	521	5429	0.89 (0.81, 0.99)
	Hispanic	10,853	1219	9634	1.08 (0.99, 1.17)
	Total	53,511	5069	48,442	−
Odds of biologic DMARD switch^a^					
Prevalent RA, prior biologic DMARD users	White, non-Hispanic	4181	532	3649	Ref
	Black, non-Hispanic	976	99	877	0.71 (0.55, 0.90)
	Other non-Hispanic	836	98	738	1.08 (0.85, 1.41)
	Hispanic	1670	192	1478	0.92 (0.76, 1.12)
	Total	7663	921	6742	−

*DMARD* disease-modifying antirheumatic drug, *RA* rheumatoid arthritis. ^a^Adjusted for demographics, data source, calendar year, comorbidities, history of medication use, and healthcare utilization in baseline period

We did not find associations between most of the comorbid conditions and initiating or switching biologic DMARDs. We observed decreased odds of biologic DMARDs initiation in patients who had early untreated RA and hypertension (OR = 0.85, 95% CI = 0.78–0.94). Having inflammatory bowel disease increased the odds of initiation of biologic DMARDs but decreased that of switching. Alcohol abuse decreased the likelihood of biologic DMARD initiation (OR = 0.77, 95% CI = 0.64–0.93) in patients with early untreated RA. However, it was not associated with biologic DMARD initiation or switching among patients with prevalent RA.

Use of coxibs and non-selective NSAIDs was significantly associated with biologic initiation among early untreated and patients with prevalent RA. Patients with early untreated RA with prior use of Cyclooxygenase inhibitors (Coxibs) were 30–46% more likely to initiate (OR = 1.46, 95% CI = 1.32–1.61) or switch biologic DMARDs (OR = 1.30, 95% CI = 0.13–1.38). Similarly, patients with early untreated RA with prior use of NSAIDs were 30% more likely to initiate a biologic DMARD (OR = 1.30 95% CI = 1.20–1.39). Opioid use was associated with both biologic DMARD initiation and switching in all three cohorts. Steroid daily dosage was strongly associated with both biologic DMARD initiation and switching. In patients with early untreated RA, use of 5–10 mg/day steroids on average increased the odds of initiating a biologic DMARD by >200% (OR = 3.12, 95% CI = 2.56, 3.80) compared to no use. Similarly, in patients with prevalent RA, medium steroid daily dosage (5–10 mg/day) also increased the odds of biologic DMARD initiation by almost 90% (OR = 1.89, 95% CI = 1.76, 2.04).

Patients with prevalent RA were more likely to initiate a biologic DMARD or switch to another biologic drug if they used more than one type of non-biologic drug during the baseline period (OR = 2.40, 95% CI = 2.17–2.67 and OR = 1.98, 95% CI = 1.65–2.36, respectively, Table 2). Prior use of MTX was associated with increased biologic DMARD initiation (OR = 1.79, 95% CI = 1.69–1.90) in patients with prevalent RA, whereas prior use of HCQ was associated with decreased biologic DMARD initiation (OR = 0.57, 95% CI = 0.54–0.61). However, both MTX and HCQ indicated decreased odds of switching to another biologic DMARD among prevalent biologic DMARD users (OR = 0.78 and 0.75, respectively).

### Patterns of switching biologic DMARDs

Etanercept was the most commonly used (51%) biologic drug during the baseline period, followed by adalimumab (22%) and infliximab (22%) (Table 4). Overall, 12% of biologic DMARD users switched to other biologic DMARDs; specifically, the largest proportion of patients switching (32.7%) among all the biologic agents was for anakinra, followed by golimumab (26.9%), with adjusted OR of 3.20 (95% CI = 2.41–4.25) and 2.24 (95% CI = 1.48–3.37), respectively (Table 4). Overall, over two thirds of patients switched from a TNF/non-TNF inhibitor to another TNF inhibitor.

**Table 4:** Switching between TNF and non-TNF biologic DMARDs

Prior biologic	Number (% of all prior biologic DMARDs)	Number of switches (% of each prior biologic DMARD)	Adjusted^a^ OR of any switch (95% CI)	Switch to TNF or to non-TNF inhibitors^b^	
				Number of switches to TNF inhibitors (%)	Number of switches to non-TNF inhibitors (%)
TNF inhibitors					
Etanercept	11,753 (50.6)	1223 (10.4)	Reference	989 (80.9)	234 (19.1)
Adalimumab	5119 (22.0)	732 (14.3)	1.22 (1.10, 1.35)	573 (78.3)	159 (21.7)
Certolizumab	104 (0.4)	20 (19.2)	1.44 (0.86, 2.39)	8 (40.0)	12 (60.0)
Golimumab	130 (0.6)	35 (26.9)	2.24 (1.48, 3.37)	22 (62.9)	13 (37.1)
Infliximab	5102 (22.0)	567 (11.1)	1.06 (0.95, 1.19)	377 (66.5)	190 (33.5)
Non-TNF inhibitor biologic agents					
Abatacept	407 (1.8)	70 (17.2)	1.31 (0.99, 1.72)	40 (57.1)	30 (42.9)
Anakinra	260 (1.1)	85 (32.7)	3.20 (2.41, 4.25)	79 (92.9)	6 (7.1)
Rituximab	334 (1.4)	27 (8.1)	0.57 (0.38, 0.85)	16 (59.3)	11 (40.7)
Tocilizumab	22 (0.1)	2 (9.1)	0.59 (0.14, 2.60)	0 (0.0)	2 (100)
Total	23231 (100)	2761 (11.9)	—	743 (76.2)	657 (23.8)

*DMARD* disease-modifying antirheumatic drug. ^a^Adjusted for demographics, data source, calendar year, comorbidities, history of medication use, and healthcare utilization in the baseline period. ^b^TNF inhibitors include adalimumab, certolizumab, etanercept, golimumab, and infliximab; non-TNF inhibitors include abatacept, anakinra, rituximab, and tocilizumab

## Discussion

Our study presents a comprehensive list of factors associated with biologic initiation or switching, and patterns of biologic DMARD use in patients with RA, based on large longitudinal databases representing both private and public sectors over the 12-year study period. We identified that initiation or switching of biologic DMARDs was associated with factors that are suggestive of history of RA treatment, such as previous use of steroids and non-biologic DMARDs. In addition, we noted that there are significant disparities in receipt of biologic treatment, by race and insurance type. For patterns of initial and subsequent choice of biologic treatment, we observed that TNF inhibitors including etanercept, adalimumab, and infliximab were the most widely used first-line and second-line biologic DMARDs, and patients were more likely to switch from golimumab or anakinra to a TNF inhibitor.

Our results showed lower odds of initiating a biologic DMARD among patients covered by public sector insurance compared to those covered by private insurance. This suggests different accessibility to biologic DMARDs under these two types of insurance programs. Fischer et al. reported that 32 out of 50 states studied had implemented or planned to implement prior authorization policies for biologic DMARDs, which may be a barrier to accessing them [10]. However, the type of health care insurance was only associated with the initial utilization of biologic DMARDs. Once RA patients had been initiated on biologic DMARDs, the insurance type did not seem to be associated with subsequent switching to other biologic DMARDs (OR = 0.92, 95% CI = 0.81–1.05).

We also found strong racial disparities in biologic DMARD utilization. Initiation of a biologic DMARD in patients with early untreated RA and in patients with prevalent RA was 40% and 30% less likely in the African-American population compared to the white population. A similar pattern was observed in previous observational studies [11–13]. We also observed that subsequent switch to another biologic DMARD in African-Americans was 30% less likely than in the white population. Disparities in biologic DMARD switching have not been reported elsewhere. Solomon et al. demonstrated that a visit to the rheumatologist acted as a mediator and after adjusting for rheumatology visit the effect of African-American race in any DMARD use was attenuated [11]. We were not able to adjust for rheumatology visit, since information on provider specialty is not available in the Medicaid database. But after adjusting for insurance type and other healthcare utilization status, we still observed a big gap by race in biologic treatment. These observed differences might be driven by differences both in accessibility to a rheumatologist and other factors, such as patients’ preferences in DMARD treatment based on benefits versus risks [13].

Our study did not find significant associations between most of the comorbidities during the baseline period and initiation/switching of biologic DMARDs among all three cohorts. However, patients with underlying metastatic cancer were less likely to switch biologic DMARDs (OR = 0.29, 95% CI = 0.10–0.81). While there is limited and conflicting evidence on the risk of cancer among biologic DMARD users, possibly due to the infrequent exposure and disease outcome [14–17], our observation suggests physicians and/or patients tend to avoid treatment with a biologic agent when patients have cancer. Use of prior medication such as Coxibs, non-selective NSAIDs, opioids, and steroids, which are probably indicative of symptoms of active RA, were positively associated with biologic DMARD initiation/switching in patients with early or untreated RA or those with prevalent RA. It suggests that before starting biologic treatment, clinicians would start with steroid treatment for patients in the early stage of the disease. Additionally, the use of non-biologic DMARDs aligned well with ACR guideline recommendations, whereby patients who had more non-biologic DMARDs before were more likely to initiate or switch biologic DMARDs due to non-response to the non-biologic treatment.

The most commonly used non-biologic drugs, MTX and HCQ, were negatively associated with biologic DMARD switching. Not switching to other biologic DMARDs may reflect better effectiveness or safety of current biologic treatment. In other words, it may support the commonly agreed fact among rheumatologists and in clinical trials that the effects of biologic DMARDs are greater in patients with RA when combined with MTX and/or HCQ [2, 18].

Etanercept, first approved by the FDA in 1988, still remains a popular biologic drug for RA treatment. It has been considered as one of the safest biologic drugs with longer treatment duration, low risk of infection, and fewer adverse events in the elderly [15, 19, 20]. Other TNF inhibitors such as adalimumab and infliximab were also used frequently with a low proportion of patients switching. Among the non-TNF biologic DMARDs, patients on anakinra had the highest odds of switching to other biologic DMARDs. It is reported in other studies that anakinra has poorer benefit and safety outcomes when compared to other biologic DMARDs [21]. Other non-TNF inhibitor biologic DMARDs, abatacept and rituximab, were unlikely to be switched when compared to etanercept. Based on 2012 ACR RA treatment recommendations, the anti-TNF biologic DMARDs, abatacept or rituximab, were recommended for initiation of a biologic DMARD after non-biologic DMARD monotherapy or combination therapy. Also, if a patient still suffered from moderate or high disease activity, then the recommendation was to change to another anti-TNF or to a non-TNF biologic DMARD [2]. Our result showed that TNF inhibitors were preferred in most circumstances, as both first-line and second-line choices. Adalimumab, etanercept, and infliximab were most common subsequent biologic DMARDs; abatacept and rituximab are used most commonly among non-TNF inhibitors as subsequent biologic DMARDs. Thus, the pattern of biologic DMARD initiation and switching in the real world setting follows the previously described ACR recommendations [2].

This study is the largest cohort study of DMARD biologic utilization patterns and trends, representing both commercially and publicly insured populations across the USA. The time span of the study covered not only the utilization of early biologic DMARDs, but also included the newer biologic DMARDs that came to the market recently. It also presented over 10 years of utilization pattern, which is not reported elsewhere in previous studies. Additionally, the United Healthcare and Medicaid databases provided comprehensive baseline characteristics of patients.

However, there are some limitations to our study. First, we acknowledge that potential misclassification of patients in the different RA groups is possible (early untreated versus prevalent). Yet, we required all patients to have continuous insurance enrollment during the study period to avoid the misclassification due to insurance switching. In addition, patients were required not to have used any DMARDs during the baseline period to be classified as having early untreated RA. It is unlikely that a patient with prevalent RA had not used any DMARD prescriptions for more than 1 year. Thus, we believe that the likelihood of RA group misclassification is minimal. Second, although our study databases provide comprehensive patient information including comorbidity, history of medication, and healthcare utilization, they still lack some clinical details such as duration or disease activity index of RA, which may have resulted in residual confounding. To minimize such confounding, we stratified the cohort into three different disease stages based on previous RA diagnosis and DMARD prescriptions and adjusted for use of analgesics and steroids, and healthcare utilization. Third, we had a limited sample size for tocilizumab and tofacitinib users as these drugs were approved more recently. This yielded wide confidence intervals for odds ratios for switching, making it hard to make any conclusions on these biologic DMARDs. Future studies are needed on the patterns of use of these newer biologic DMARDs. Last, racial/ethnicity data were limited to the Medicaid population. Therefore, our findings on racial disparities may not be generalizable to commercially insured patients.

## Conclusions

This large longitudinal cohort study of RA patients enrolled in either a public or private health plan indicated important factors that are associated with biologic DMARD utilization patterns. Notably, we demonstrated potential disparities in access to biologic DMARDs in the early phases of RA by insurance status and race. Future educational interventions aimed at addressing these disparities are warranted to ensure equitable access to biologic medications and achieve optimal disease control in RA.

## Abbreviations

ACE: Angiotensin-converting enzyme
ACR: American College of Rheumatology
ARB: Angiotensin II receptor blockers
CI: Confidence interval
COPD: Chronic obstructive pulmonary disease
Coxibs: Cyclooxygenase inhibitors
DMARD: Disease-modifying antirheumatic drugs
FDA: Food and Drug Administration
HCQ: Hydroxychloroquine
ICD-9: International Classification of Disease, Ninth Revision
MAX: Medicaid Analytic eXtract
MTX: Methotrexate
NSAIDS: Non-selective non-steroidal anti-inflammatory drugs
OR: Odds ratio
RA: Rheumatoid arthritis
TNF: Tumor necrosis factor
